# Corneal protective effects of a new ophthalmic formulation based on vitamin B12 and sodium hyaluronate

**DOI:** 10.3389/fphar.2025.1548213

**Published:** 2025-01-31

**Authors:** Francesca Lazzara, Federica Conti, Grazia Maugeri, Velia D’Agata, Ludovica Sotera, Claudio Bucolo

**Affiliations:** ^1^ Department of Biomedical and Biotechnological Sciences, School of Medicine, University of Catania, Catania, Italy; ^2^ Center for Research in Ocular Pharmacology-CERFO, University of Catania, Catania, Italy

**Keywords:** cornea, dry eye disease, vitamin B12, sodium hyaluronate, trehalose

## Abstract

**Introduction:**

Corneal damage can happen due to a variety of insults, including environmental factors and iatrogenic issues. For instance, the corneal epithelium is sensitive to oxidative stress caused by reactive oxygen species (ROS) or by ultraviolet B (UVB) radiation. Moreover, the strictly correlated oxidative damage and inflammatory processes impair the corneal reparative wound healing mechanism. Corneal protection after damage remains an unmet medical need that requires urgent management. Sodium hyaluronate is known to protect the cornea against oxidative and inflammatory injury. Additionally, vitamin B12 is a good candidate for counteracting corneal damage, helping preserve visual functions.

**Methods:**

The present study aimed to investigate the potential protective effect of an ophthalmic formulation based on 0.01% vitamin B12% and 0.15% sodium hyaluronate (DROPYAL EVO) compared to other ophthalmic formulations containing sodium hyaluronate and trehalose (TRIMIX and THEALOZ DUO). Two different *in vitro* models of corneal damage were carried out in corneal epithelial cells exposed to hydrogen peroxide (H_2_O_2_, 1 mM) or UVB (20 mJ/cm^2^). Cell viability, cytotoxicity, ROS production, and mRNA expression of pro-inflammatory cytokines (TNF-α and IL-1β) were assessed by MTT, LDH, 2′,7′–dichlorofluorescein diacetate (DCFDA) assays and Real-time PCR, respectively. Additionally, the ability of ophthalmic formulations to affect the wound healing process in corneal epithelial cells was assessed at different time points by scratch wound healing assay.

**Results:**

The eye drops containing vitamin B12 were able to significantly counteract oxidative and inflammatory damage in corneal epithelial cells exposed to H_2_O_2_ stimulus and UVB radiation, in terms of ROS production and pro-inflammatory cytokines expression. Additionally, the eye drops containing vitamin B12 obtained significantly better outcomes in terms of wound closure at 36 h and 48 h after scratching the corneal epithelial cells, compared to the other two formulations containing trehalose.

**Discussion:**

Vitamin B12 potentially enhances the protective effect of sodium hyaluronate, accelerating the wound healing process and modulating oxidative stress and inflammation. Vitamin B12, in combination with sodium hyaluronate, could represent a promising approach to managing corneal epithelial damage. Further clinical investigations are needed to confirm this data.

## 1 Introduction

The cornea is a transparent and avascular eye tissue with the dual function of enclosing and protecting the inner structures of the eye. The corneal epithelium is the main barrier to pathogens and noxious stimuli (Suzuki et al., 2003; Eghrari et al., 2015). Corneal tissues can be injured due to surgical and non-surgical conditions (Romano et al., 2014; Sepulveda-Beltran et al., 2022; Alves et al., 2023), such as cataract surgery, corneal transplantation, and different chemical, physical, and pathological insults, representing an important medical issue requiring management (Ljubimov and Saghizadeh, 2015; Bandeira et al., 2019). Additionally, the eye is particularly susceptible to oxidative stress due to its anatomical localization and the high metabolic activities of its structures. This can lead to increased ocular surface damage by producing reactive oxygen species (ROS) (Böhm et al., 2023).

One of the main exogenous factors contributing to ROS production in the eye is solar ultraviolet (UV) radiation, which plays a role in the pathogenesis of several eye disorders (Balasubramanian, 2000; Brennan and Kantorow, 2009; Marchitti et al., 2011). UV radiation also promotes hydrogen peroxide (H_2_O_2_) production, which damages DNA, lipids, and proteins, ultimately resulting in tissue injury (Marchitti et al., 2011). H_2_O_2_ is one of the main non-radical ROS and is ubiquitously produced in different ocular tissues, such as the humor aqueous, cornea, and surrounding tissues (Hudde et al., 2002; Izzotti et al., 2009). H_2_O_2_ has a prominent role as a damage signal molecule, and is strictly correlated with the generation of toxic hydroxylic radicals (Lushchak, 2014; Halliwell et al., 2021). Moreover, H_2_O_2_ can activate different inflammatory pathways, leading to overexpression of pro-inflammatory cytokines like IL-1β and TNF-α and exacerbating oxidative stress and ROS production (Bucolo et al., 2018; Al Sabaani, 2020; Lazzara et al., 2021; Wang Q. et al., 2023).

Oxidative stress is a hallmark of different corneal diseases (Kruk et al., 2015), contributing to the development and progression of ocular surface pathologies, such as dry eye disease (DED). DED is a multifactorial disorder characterized by a deficiency in tear production or excessive tear evaporation, inflammation, and corneal epithelial lesions (Navel et al., 2022; Böhm et al., 2023; Chu et al., 2024). Based on the premises above, anti-inflammatory and anti-oxidant compounds may represent an option to counteract corneal damage in DED and other ocular conditions (Anfuso et al., 2017; Bucolo et al., 2018; 2023).

Topical vitamin B12 treatment has shown anti-oxidant and anti-inflammatory effects, protecting the eye from ROS-induced damage and maintaining a healthy ocular surface (Macri et al., 2015; Yang et al., 2019; De-Hita-Cantalejo et al., 2022). In addition, vitamin B12 restores the integrity of the cornea after mechanical injury, accelerating corneal re-epithelization and re-innervation (Romano et al., 2014; Bucolo et al., 2023). Conversely, vitamin B12 deficiency has been associated with severe DED and neuropathic ocular pain (Ozen et al., 2017), optic neuropathy (Chavala et al., 2005), eye movement disorders (Akdal et al., 2007) and corneal epitheliopathy (Jurkunas et al., 2011). On the other hand, sodium hyaluronate is used during intraocular surgeries to protect the corneal endothelium and stabilize the anterior chamber (Graue et al., 1980; Higashide and Sugiyama, 2008) due to its viscoelastic properties. Sodium hyaluronate also prompts corneal epithelial cells’ healing by stimulating their migration, adhesion, and proliferation (Nishida et al., 1991; Gomes et al., 2004). It is also used as a treatment for DED, to maintain a healthy corneal epithelium (Shimmura et al., 1995). We hypothesized that combining vitamin B12 and sodium hyaluronate would reduce ROS and inflammation and improve wound healing in corneal epithelial cells after inducing H_2_O_2_ or UV radiation exposure damage. Therefore, our aim here was to assess the potential protective effect of 0.01% vitamin B12% and 0.15% sodium hyaluronate-based ophthalmic formulation (DROPYAL EVO) in comparison to two formulations containing sodium hyaluronate and trehalose (TRIMIX and THEALOZ DUO) using Statens Seruminstitut Rabbit Corneal (SIRC) epithelial cells as *in vitro* models of the damaged corneal epithelium.

## 2 Materials and methods

### 2.1 Ophthalmic formulations

Three different ophthalmic formulations were investigated: formulation A, DROPYAL EVO (0.15% sodium hyaluronate, 0.01% Vit.B12; Bruschettini S.r.l, Genova, Italy); formulation B, TRIMIX (0.15% cross-linking sodium hyaluronate, 3% trehalose; Off Health, Florence Italy) and formulation C, THEALOZ DUO (0.15% sodium hyaluronate, 3% trehalose; Laboratoires Théa, Clermont-Ferrand, France). All the formulations were used at 5% v/v (% volume/volume) by dilution in cell medium. A 30-min pre-treatment was carried out for all experiments.

### 2.2 Cell culture and experimental groups

SIRC cells, obtained from the American Type Culture Collection (ATCC^®^, Manassas, Virginia, United States), were cultured in Eagle’s Minimum Essential Medium (ATCC^®^ 30-2003, Manassas, Virginia, United States) with 10% activated fetal bovine serum (FBS, 12103C, Sigma-Aldrich, St Louis, MO, United States) and incubated at 37°C in a humidified atmosphere of 5% CO_2_ as previously described (Maugeri et al., 2022). Experimental groups: negative control, positive control, group A: DROPYAL EVO 5%, group B: TRIMIX 5%; group C: THEALOZ 5%. SIRC cells were subjected to two different oxidative stress challenges, as well as hydrogen peroxide (H_2_O_2_) treatment or exposure to ultraviolet B radiations (UV-B). In the first model, cells were cultured in a control medium (CTRL, negative control) or treated with 1 mM H_2_O_2_ (Sigma-Aldrich, St Louis, MO, United States) for 1 h or 6 h (positive control), or pre-incubated with 5% of DROPYAL EVO (A), TRIMIX (B), or THEALOZ (C) formulations for 30 min before H_2_O_2_ treatment. In the second model, SIRC cells were cultured in a control medium (CTRL, negative control) or subjected for 30 s to UV irradiation (positive control) at a dose of 20 mJ/cm^2^ by using a UV-B lamp at 302 nm with a filter size of 21 cm × 26 cm (Uvitec, Cambridgeshire, UK), or pre-incubated with 5% of A, B or C formulations for 30 min before the UV-B insult, and then kept in culture for 24 h (Maugeri et al., 2020).

### 2.3 Cell viability assay

Cell viability was assessed using the 3-[4,5-dimethylthiazol-2-y l]-2,5-diphenyl tetrasodium bromide (MTT)-based colorimetric assay (Sigma-Aldrich, St Louis, MO, United States). Cells were cultured into 96-well plates at a density of 1 × 10^4^ cells/well in 100 μL of the culture medium (Maugeri et al., 2021). After overnight growth, SIRC cells were grown according to the previously described experimental conditions. At the end of treatments, the culture medium was replaced with fresh medium with 0.5 mg/mL of MTT salt for 3 h. The reaction was stopped by adding 100 μL of dimethyl sulfoxide (DMSO). The formazan formed by the cleavage of the yellow tetrazolium salt MTT was measured spectrophotometrically by an absorbance change at 570 nm in a plate reader (VariosKan, Thermo Fisher Scientific, Waltham, MA, United States). Six independent experiments were performed, each with six replicates for each group. The medium alone was used as a blank.

### 2.4 LDH cytotoxicity assay

Cellular cytotoxicity was evaluated using the Invitrogen CyQUANT LDH Cytotoxicity Assay kit (C20300, Thermo Fisher Scientific, United States) as previously described (Lazzara et al., 2024). Briefly, SIRC cells were seeded into 96-well plates at a density of 1 × 10^4^ cells/well. After overnight growth, SIRC cells were grown in the previously described experimental conditions. At the end of treatments, 50 µL of the medium was transferred into a new 96-well plate, and a volume of 50 µL of the working solution was added. After 30 min at room temperature, 50 µL of the stop solution was added. The absorbance was measured with the Varioskan microplate reader at 490 nm and 680 nm (Thermo Fisher Scientific, Waltham, MA, United States). LDH activity was determined by subtracting the 680-nm absorbance value (background) from the 490-nm absorbance. The % cytotoxicity was calculated by using the following formula:

$$Citotoxicity\;\left(\%\right)=\frac{Abs_{x}}{Abs_{ctrl}+}\;x100$$

where Abs_x_ is the absorbance in the x well, and Abs_ctrl+_ is the average absorbance of internal positive control cells (untreated lysed cells). Six replicate wells were used for each group. Absorbance values were corrected by subtracting blanks.

### 2.5 Detection of ROS

ROS levels were assessed using the 2′,7′–dichlorofluorescein diacetate (DCFDA)–Cellular ROS Detection Assay Kit (ab113851, Abcam, Cambridge, UK), as previously described (Lazzara et al., 2024). SIRC cells were plated into 96-well black plates at a density of 1 × 10^4^ cells/well for 24 h. After overnight growth, SIRC cells were cultured in different experimental conditions. At the end of treatments, cells were washed twice gently with PBS and incubated with 25 μM DCFDA. The ROS concentration was determined by measuring the DCF fluorescence (λ_ex_ = 495 nm, λ_em_ = 529 nm) with the Varioskan microplate (Thermo Fisher Scientific, Waltham, MA, United States). Twelve replicate wells were used for each group.

### 2.6 Scratch wound healing assay

SIRC cells (0.3 × 10^6^) were seeded in a 6-well plate and grown in a complete medium until 90%–95% confluence. When confluence was reached, cells were washed twice with PBS (1x) and then incubated with serum-free medium for 5 h. A disposable pipette tip (200 μL volume) was used to scratch wounds on the midline of the well, which was carefully washed with the complete medium to remove floating cells and clean the wound area. Then, cells were incubated in a serum-free medium alone (CTRL) or with 5% of A, B or C formulations for 72 h. Pictures of the wound areas were taken, and the coordinates noted at the starting time of the experiment (T0) and then 12 h (T12), 24 h (T24), 36 h (T36), 48 h (T48) and 72 h (T72) after the scratch. T0, T12, T24, T36, T48 and T72 wound images were acquired with a Leica microscope (×10 magnification). The average wound area, expressed in the percentage of control (CTRL), was determined using ImageJ Software (Broken Symmetry Software, Bethesda, MD, US).

### 2.7 Extraction of total ribonucleic acid and cDNA synthesis

Total RNA extraction from SIRC cells was performed with a TRIzol Reagent (Invitrogen, Life Technologies, Carlsbad, CA, United States). The A_260_/A_280_ ratio of optical density of RNA samples (measured with Multimode Reader Flash Varioskan™, Thermo Fisher Scientific, Waltham, MA, United States) was 1.95–2.01; this RNA purity was confirmed with the electrophoresis in non-denaturing 1% agarose gel (in TAE). cDNA was synthesized from 2 µg RNA with a reverse transcription kit (SuperScript™ II Reverse transcriptase, Invitrogen, Thermo Fisher Scientific, Carlsbad, CA, United States).

### 2.8 Real-time PCR

Real-time PCR was carried out with the Rotor-Gene Q (Qiagen, Milan, Italy). The amplification reaction mix included the Master Mix Qiagen (10 µL) (Qiagen QuantiNova SYBR Green Real-Time PCR Kit, Milan, Italy) and cDNA (1 μL, 100 ng). Forty-five amplification cycles were carried out for each sample. Results were analyzed with the 2^−ΔΔCt^ method. Quantitative PCR experiments followed the MIQE guidelines (Bustin et al., 2009). Gene expression levels were normalized with levels of the housekeeping genes (HPRT, hypoxanthine phosphoribosyl transferase). Primers were purchased from Eurofins Genomics (Milan, Italy) and Qiagen (Milan, Italy). Forward and reverse primer sequences (for rabbit genes) were: IL-1β (forward: 5′-GCC​TCA​GGG​GGA​AGA​ATC​TG -3'; reverse: 5′-TGG​GGT​CTA​CAC​TCT​CCA​GC -3′), TNF-α (forward 5′-GGA​GCT​GCC​TTG​GTT​CTC​AC -3'; reverse 5′-ATG​TAG​CGA​CGG​GTC​AGT​CA -3′), HPRT (forward 5′-ACA​GGC​CAG​ACT​TTG​TTG​GA -3'; reverse 5′-ACT​GGC​GAT​GTC​AAT​GAG​ACT -3′).

### 2.9 Statistical analysis

Statistical analysis was performed using GraphPad Prism 10 (GraphPad, La Jolla, California). The data generated by all experiments are reported as mean ± SD. A one-way analysis of variance (ANOVA) was carried out, and Tukey’s *post hoc* test was used to make multiple comparisons. Differences between groups were considered statistically significant for p-values <0.05.

## 3 Results

### 3.1 Effects of ocular formulations against oxidative stress

**FIGURE 1:**
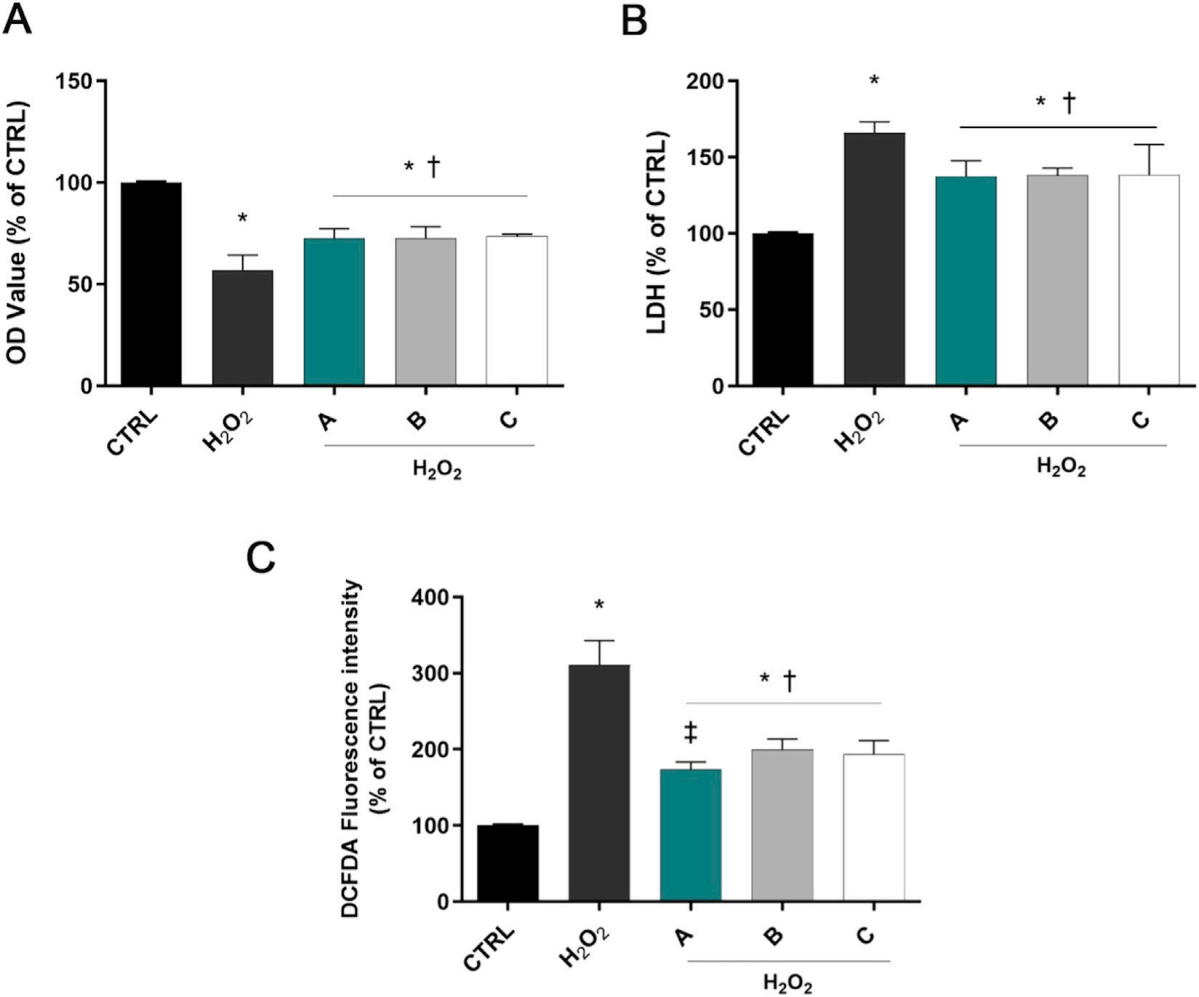
Formulations counteracted H_2_O_2_ cell damage. Cells were pretreated for 30 min with 5% formulations DROPYAL EVO (A), TRIMIX (B) or THEALOZ (C) and for 1 h with H_2_O_2_ insult (1 mM). At the end of treatment MTT **(A)**, LDH **(B)**, and the ROS assay **(C)** were carried out. Values are reported as mean ± SD (from n = 6 independent experiments). Data were analyzed by one-way ANOVA and Tukey’s *post hoc* test for multiple comparisons. *p < 0.05 vs. control; †p < 0.05 vs. H_2_O_2_; ‡p < 0.05 vs. B, C.

As shown in Figure 1A, 1 h incubation with H_2_O_2_ at 1 mM decreased cell viability compared with control cells (p < 0.05 vs. CTRL). SIRC cells pretreated with A, B, or C formulations significantly increased cell viability compared to H_2_O_2_-treated cells (p < 0.05 vs. H_2_O_2_). Similarly, LDH activity was increased by H_2_O_2_ treatment, but significantly decreased by pre-treatment with the three different formulations, confirming their ability to counteract H_2_O_2_-induced corneal epithelial cell damage (p < 0.05 vs. H_2_O_2_, Figure 1B).

ROS levels increased in H_2_O_2_-treated cells compared to controls (p < 0.05 vs. CTRL, Figure 1C). However, pre-treatment with the three different formulations prominently decreased the fluorescence intensity compared to the H_2_O_2_-only group (p < 0.05 vs. H_2_O_2_). Noteworthy, pre-treatment with DROPYAL EVO (A) outperformed the other formulations (p < 0.01 vs. B, C).

**FIGURE 2:**
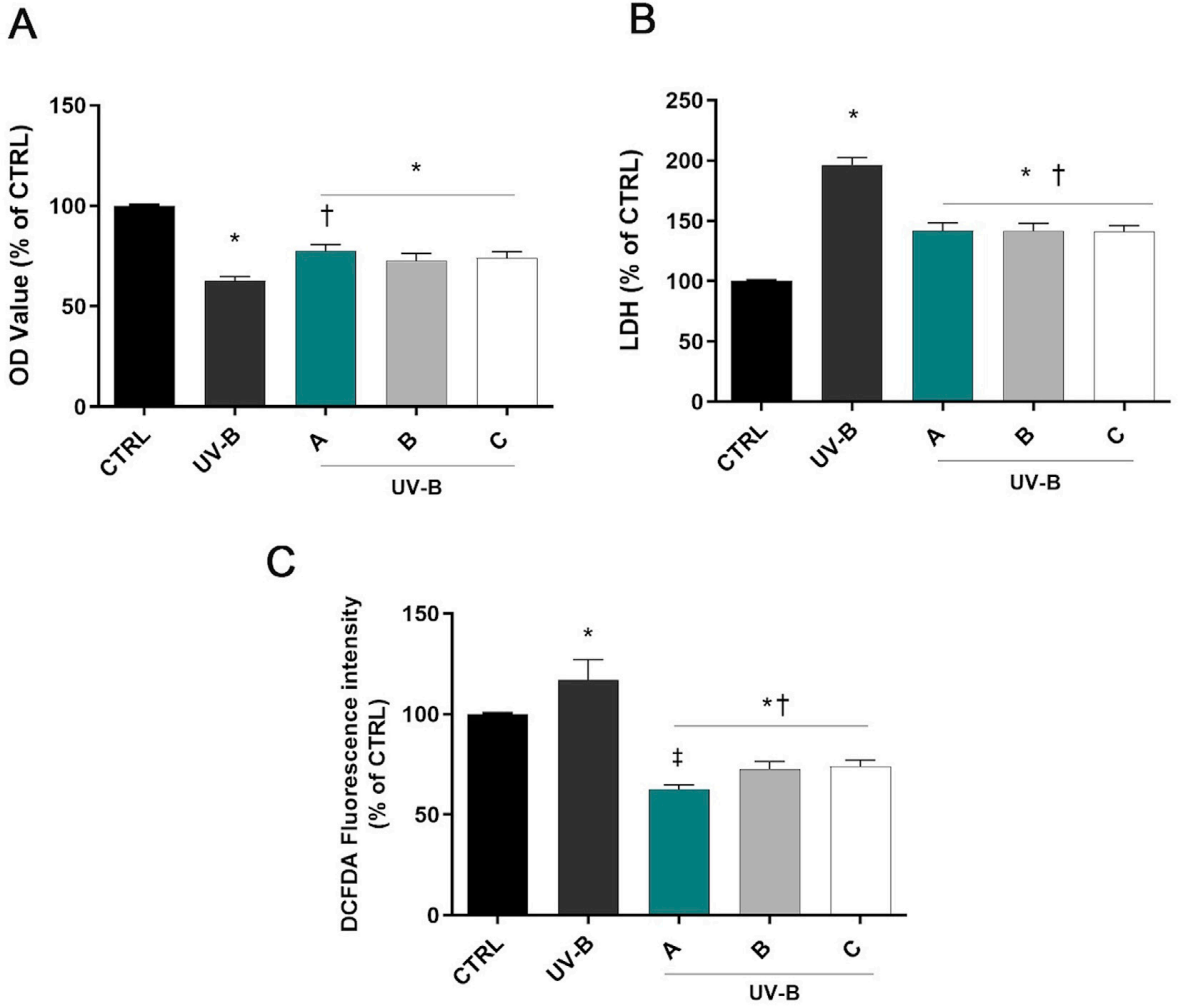
Formulations counteracted UV-B cell damage. Cells were pretreated for 30 min with 5% formulations DROPYAL EVO (A), TRIMIX (B) or THEALOZ (C) and exposed to UV-B insult for 30 s at a dose of 20 mJ/cm^2^ and then kept in culture for 24 h. At the end of treatment out MTT **(A)**, LDH **(B)**, and the ROS assay **(C)** were carried out. Values are reported as mean ± SD (from n = 6 independent experiments). Data were analyzed by one-way ANOVA and Tukey’s *post hoc* test for multiple comparisons. *p < 0.05 vs. control; †p < 0.05 vs. H_2_O_2_; ‡p < 0.05 vs. B, C.

Figure 2A shows a significant decrease in cell viability in SIRC cells exposed to UV-B for 30 s (p < 0.05 vs. CTRL). However, pre-treatment with the three different formulations significantly increased cell viability (p < 0.05 vs. UV-B). Moreover, SIRC cells exposed to UV-B showed a significant increase in the LDH release in the culture medium, indicating the occurrence of cell death due to UV-B exposure (Figure 2B). On the other hand, the corneal epithelial cells pretreated with A, B, or C formulations showed significantly reduced LDH concentration compared to UV-B treated cells (p < 0.05 vs. UV-B). Regarding intracellular ROS, a significant increase of ROS levels was observed after UV-B radiations (p < 0.05 vs. CTRL). However, pre-treatment with A, B, or C formulations significantly reduced ROS levels (p < 0.05 vs. UV-B). Remarkably, DROPYAL EVO A reduced intracellular ROS to a larger extent than the other formulations (p < 0.01 vs. B, C, Figure 2C).

### 3.2 Effect of ocular formulations on the modulation of inflammatory cytokines (IL-1β and TNF-α) after H_2_O_2_ challenge

**FIGURE 3:**
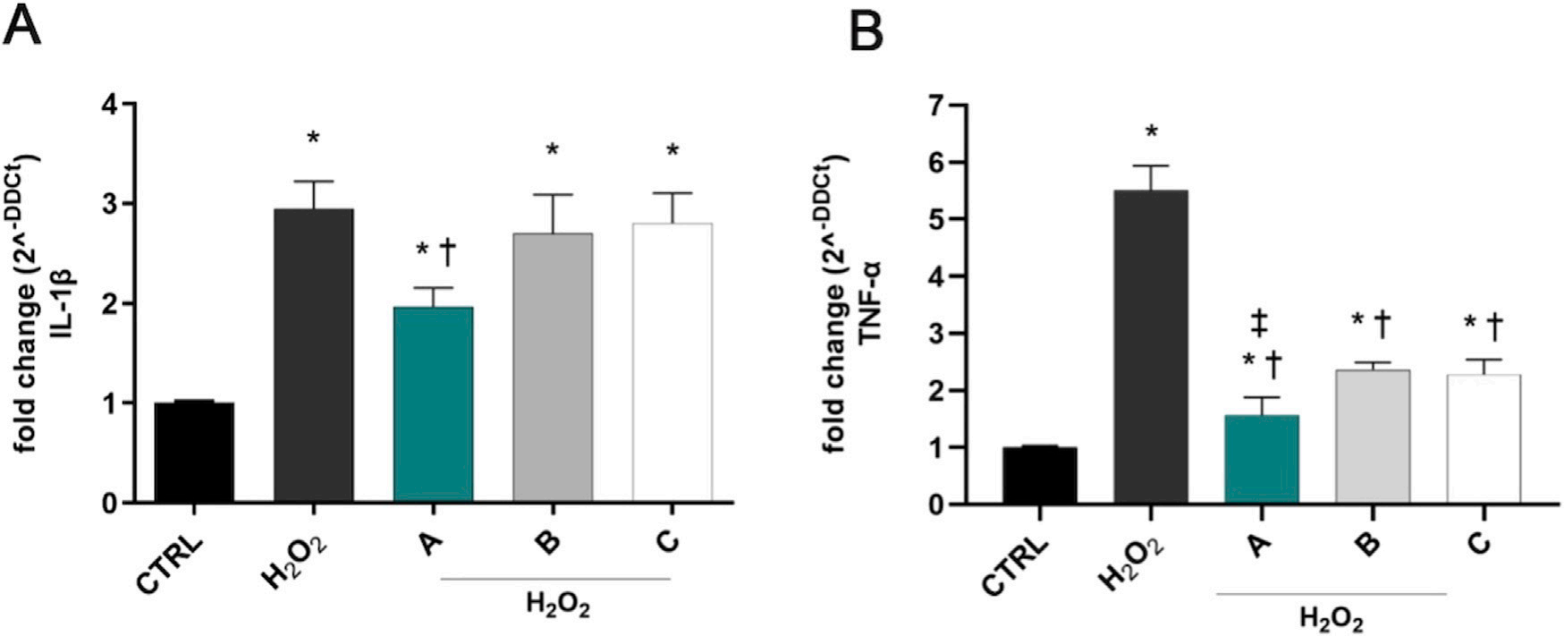
Formulations reduced the overexpression of IL-1β and TNF-α in SIRC cells, induced by H_2_O_2_ stimulus. IL-1β **(A)** and TNF-α **(B)** mRNAs were upregulated in SIRC cells after 1 mM H_2_O_2_ treatment for 6 h. Pre-treatment with 5% formulation DROPYAL EVO (A) significantly reduced mRNA levels of both cytokines, in comparison to only H_2_O_2_ treated cells and TRIMIX (B) and THEALOZ (C) treated cells. Values are reported as mean ± SD (from n = 6 independent experiments). Data were analyzed by one-way ANOVA, and Tukey post-hoc test for multiple comparisons. *p < 0.05 vs. control; † p < 0.05 vs. H_2_O_2_ and B, C; ‡ p < 0.05 vs. B, C.

H_2_O_2_ exposure induced a significant (p < 0.05 vs. CTRL) overexpression of IL-1β and TNF-α in SIRC cells after 6 h (Figure 3). DROPYAL EVO significantly decreased IL-1β (Figure 3A) and TNF-α (Figure 3B) mRNA not only in comparison to the positive control cells but also in comparison to the other formulations (p < 0.05 vs. B, C).

### 3.3 Effect of formulations on the modulation of inflammatory cytokines (IL-1β and TNF-α) after UV-B challenge

**FIGURE 4:**
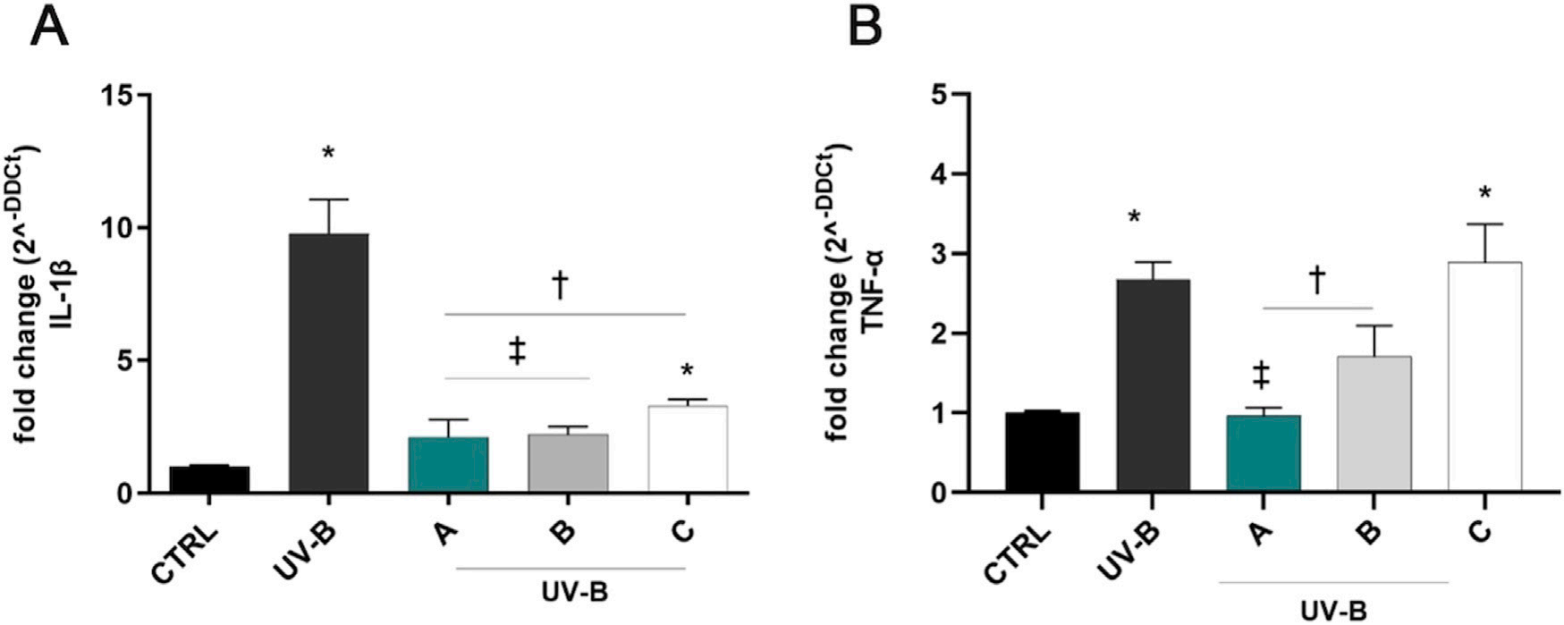
Formulations counteracted IL-1β and TNF-α overexpression induced by UV-B damage in SIRC cells. IL-1β **(A)** and TNF-α **(B)** mRNAs were upregulated in SIRC cells after UV-B exposure for 24 h. Pre-treatment with 5% formulation DROPYAL EVO (A) significantly reduced both mRNA levels in comparison to UV-B exposed cells. DROPYAL EVO (A) reduced IL-1β mRNA in comparison to THEALOZ (C) treated cells **(A)** and TNF-α mRNA compared to TRIMIX (B) and THEALOZ (C) treated cells **(B)**. Values are reported as mean ± SD (from n = 6 independent experiments). Data were analyzed by one-way ANOVA, and Tukey post-hoc test for multiple comparisons. *p < 0.05 vs. control; † p < 0.05 vs. UV-B, ‡ p < 0.05 vs. C **(A)** and vs. B, C **(B)**.

UV-B exposure induced a significant (p < 0.05 vs. CTRL) upregulation of IL-1β and TNF-α mRNAs after 24 h of the challenge (Figure 4). Notably, pre-treatment for 30 min with the three formulations counteracted the UV-B-induced damage, significantly reducing IL-1β mRNA levels in comparison to positive control cells (UV-B, Figure 4A). DROPYAL EVO and TRIMIX treatments were more effective in reducing IL-1β mRNA levels than THEALOZ (p < 0.05 vs. C). Regarding TNF-α, both DROPYAL EVO and TRIMIX formulations reduced the expression of TNF-α (p < 0.05 vs. UV-B), unlike THEALOZ. DROPYAL EVO was superior to TRIMIX and THEALOZ (p < 0.05 vs. B, C), suppressing the expression of TNF-α mRNA (Figure 4B).

### 3.4 Effect of ocular formulations on SIRC cells wound repair

**FIGURE 5:**
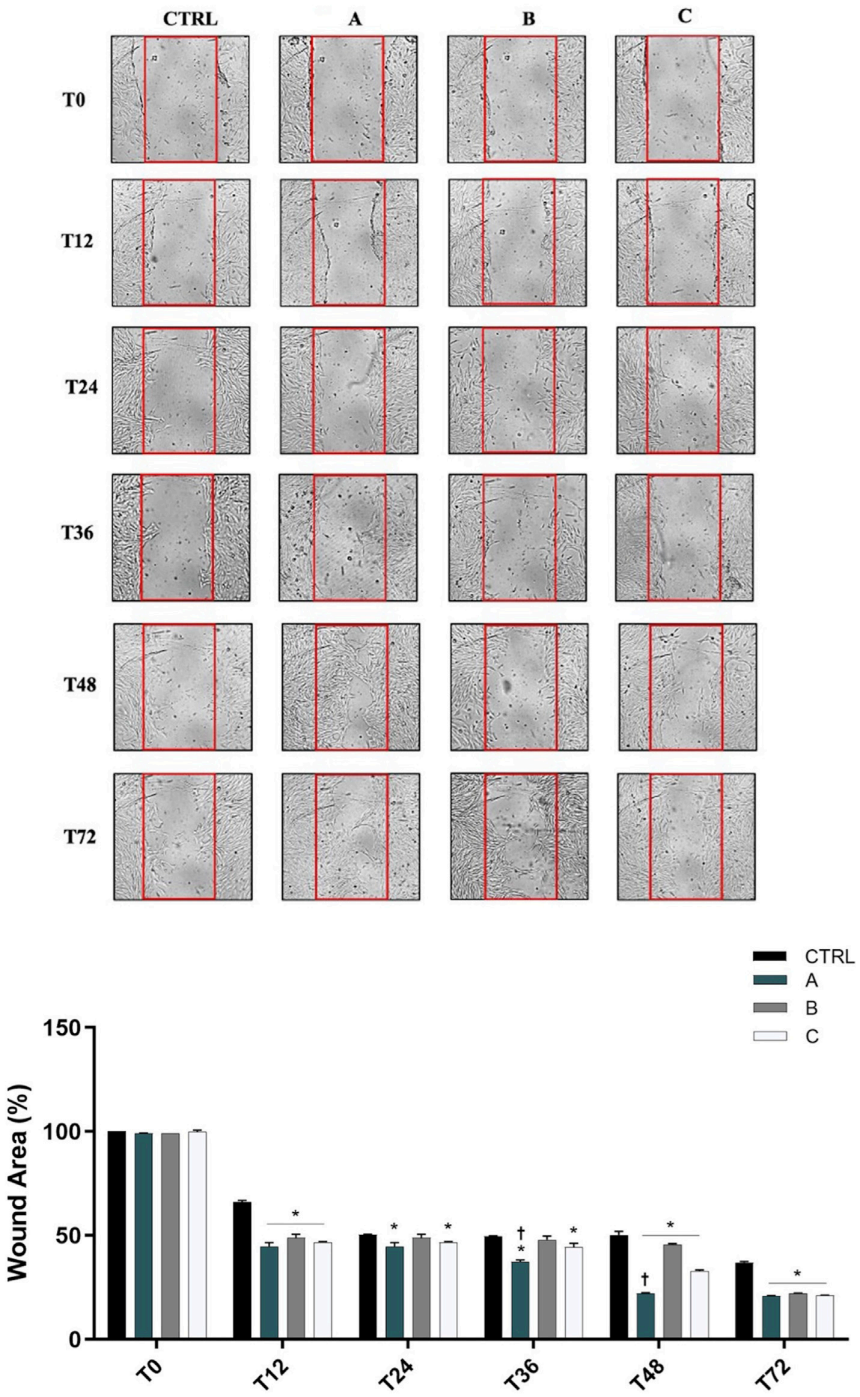
Formulations promoted wound closure in SIRC cells. (Top Panel) Representative images of wound healing assays performed in SIRCs exposed to the three different formulations (5% formulations DROPYAL EVO (A), TRIMIX (B) or THEALOZ (C) at 0, 12, 24, 36, 48 and 72 h. (Bottom Panel). The bar graph shows the average wound area expressed in the percentage of CTRL. Data are shown as mean ± SD (from n = 6 independent experiments). Data were analyzed by one-way ANOVA and Tukey’s *post hoc* test for multiple comparisons. *p < 0.01 vs. CTRL; †p < 0.05 vs. B, C.

As shown in Figure 5 12 h after confluent SIRC cells were scratched, all formulations produced a significant (p < 0.05 vs. CTRL) reduction of the average wound area compared to the control condition. However, from 24 h until 36 h, only A and C formulations showed a significant reduction of wound area (p < 0.05 vs. CTRL). Starting from 48 h until the end of the experiment (72 h), all formulations were shown to significantly promote wound healing closure (p < 0.05 vs. CTRL). Notably, DROPYAL EVO was superior in terms of wound closure than the other formulations at 36 h and 48 h (p < 0.01 vs. B, C).

## 4 Discussion

The eye is a relatively unprotected organ usually exposed to environmental chemicals, physical abrasion, pathological stimuli, atmospheric oxygen and solar UV radiation. Specifically, as the outermost layer of the eye, the cornea is particularly sensitive to external insults and environmental stressors, resulting in frequent damage (DelMonte and Kim, 2011). These external environmental factors may trigger pathological changes in corneal morphology, leading to several corneal diseases, which can potentially cause corneal blindness, unless they are adequately treated (Um et al., 2014; Osae et al., 2017; Song et al., 2018; Ma et al., 2021). When the outermost epithelium cell layer of the cornea is compromised, the epithelial wound healing process is essential to restore the normal multilayered structure of the epithelium, which is crucial to maintaining the corneal barrier function and its refractive surface (Bukowiecki et al., 2017).

Corneal wound healing represents a dynamic and complex mechanism involving overlapping phases, such as inflammation, proliferation, matrix deposition and remodeling (Rodrigues et al., 2019; Wang et al., 2022). This process implicates the cooperation between different cell types, and it is regulated by several growth factors, cytokines, and extracellular matrix proteins (Ljubimov and Saghizadeh, 2015; Rodrigues et al., 2019). Nevertheless, sustained inflammation can delay the healing process by blocking the remodeling stage, consequently leading to chronic non-healing wounds (Pierce, 2001; Goldberg et al., 2007; Wang et al., 2022). Excessive oxidative stress can negatively affect the wound healing process (Wang G. et al., 2023). Although antioxidant compounds typically help accelerate epithelial wound healing (Foresti et al., 2015; Bucolo et al., 2023; Li et al., 2023), their effectiveness may be compromised under sustained oxidative conditions. Therefore, there is an urgent need to develop efficient ways to prevent prolonged corneal inflammation and oxidative stress in order to enhance the corneal healing process and mitigate corneal injury (Bucolo et al., 2023; Zhao et al., 2023).

Sodium hyaluronate, a natural glycosaminoglycan of the extracellular matrix, is known to promote corneal epithelial wound healing (Inoue and Katakami, 1993; Gomes et al., 2004). Moreover, its topical application on the ocular surface protects the cornea from epithelial damage, for example, during intraocular surgery (Aragona et al., 2002; Hynnekleiv et al., 2022). Therefore, sodium hyaluronate is one of the components of tear substitutes for the treatment of DED.

Vitamins are essential for corneal health, and an imbalance can adversely affect corneal function and lead to diseases (Vitar et al., 2022; Bucolo et al., 2023). Particularly, vitamin B12 deficiency has been correlated with corneal epitheliopathy, optic neuropathy, eye movement disorders, corneal pain and DED symptoms (Ozen et al., 2017; Ata et al., 2020). Moreover, increasing evidence suggests an emerging role of vitamin B12 in the modulation of oxidative stress and inflammation (Gopinath et al., 2013; Hughes et al., 2013; Mikkelsen and Apostolopoulos, 2018; van de Lagemaat et al., 2019; Simonenko et al., 2024). A randomized controlled clinical trial found that patients with DED who were treated with eye drops containing hyaluronic acid and vitamin B12 experienced reduced levels of oxidative stress (Macri et al., 2015). Another trial showed an increase of basal epithelial cell density in DED patients after its ocular nebulization (Yang et al., 2019). In addition, vitamin B12 in combination with hyaluronic acid improves dry eye symptoms in menopausal women and DED patients (Macri et al., 2015; De-Hita-Cantalejo et al., 2022).

Based on these premises, the present study compared DROPYAL EVO, which contains 0.01% vitamin B12% and 0.15% sodium hyaluronate, to TRIMIX and THEALOZ DUO, both of which only contain sodium hyaluronate and trehalose. The evaluation was conducted using two *in vitro* models of corneal damage: the H_2_O_2_ challenge and UVB radiation exposure.

All three tested formulations were able to counteract H_2_O_2_/UVB-induced ROS production, confirming the protective effects of sodium hyaluronate by reducing ROS production in corneal epithelial cells (Pauloin et al., 2008; 2009; Wu et al., 2011; Li et al., 2013; Bucolo et al., 2018). Notably, however, among the three formulations, DROPYAL EVO showed the best result in ROS reduction in both *in vitro* models, which can be attributed to the presence of vitamin B12. In line with this finding, a recent study in the same model have shown that a new formulation containing vitamin B12 was the most effective at reducing ROS production (Bucolo et al., 2023).

Pathological damage to the corneal epithelium can induce sustained inflammatory processes, which may impair wound healing, ultimately altering corneal structures and leading to visual impairment (Zuo et al., 2019; Shu et al., 2023). In particular, inflammatory processes could be stimulated by oxidative stress damage (Biswas, 2016). H_2_O_2_ can activate NF-κβ inflammatory signaling pathway, in turn, stimulating the overexpression of pro-inflammatory cytokines, such as TNF-α and IL-1β, in corneal epithelial and endothelial cells, and in retinal and lens epithelial cells (Dudek et al., 2001; Lazzara et al., 2021; Wang Q. et al., 2023). Similarly, UVB radiation stimulates an inflammatory response in the corneal epithelium, inducing the secretion of pro-inflammatory cytokines (Black et al., 2011; Maugeri et al., 2023). Accordingly, we observed that both H_2_O_2_ and UVB radiation exposure elicited the overexpression of IL-1β and TNF-α in corneal epithelial cells, stimulating an inflammatory response. Notably, the DROPYAL EVO formulation was able to counteract the overexpression of these inflammatory cytokines to a larger extent than the other formulations, which could be partly attributed to the anti-inflammatory properties of vitamin B12 (Halczuk et al., 2023). An *in vitro* study showed an increased gene expression of pro-inflammatory cytokines, including IL-1β and TNF-α, in human adipocytes cultured in low vitamin B12 conditions (Samavat et al., 2018), and a vitamin B12 analogue suppressed inflammatory cytokines of T lymphocytes (Yamashiki et al., 1992). Moreover, decreased serum levels of vitamin B12 in aged mice, as well as in patients with high cardiovascular risk or with diabetes, have been correlated with increased inflammatory markers, suggesting again an anti-inflammatory effect of this vitamin (Lee et al., 2016; Domínguez-López et al., 2024).

Lastly, regarding corneal epithelial wound closure, DROPYAL EVO achieved the best outcome in terms of wound closure at 36 h and 48 h compared to TRIMIX and THEALOZ DUO. This result aligns with previous reports showing the role of vitamin B12 in prompting wound healing in the corneal epithelium and in stimulating re-epithelization after corneal injury in different animal models (Romano et al., 2014; Bucolo et al., 2023). No side effects are reported for topical vitamin B12 in ocular surface (Fogagnolo et al., 2021); incidentally, the ophthalmic formulation containing vitamin B12 (DROPYAL EVO) is already approved for clinical use. Further studies will be useful to evaluate the contribution of vitamin B12 in terms of corneal wound healing in clinical practice. In conclusion, our results support the hypothesis that the combination of vitamin B12 and sodium hyaluronate protects corneal epithelial cells from oxidative and inflammatory damage, prompting the corneal wound healing process.

## Data Availability

The raw data supporting the conclusions of this article will be made available by the authors, without undue reservation.
